# Intraoperative ultrasonography in laminectomy for degenerative cervical spondylotic myelopathy: a clinical and radiological evaluation

**DOI:** 10.1007/s00701-022-05232-8

**Published:** 2022-05-10

**Authors:** Annamaria Biczok, Manuel Fuetsch, Sebastian Siller, Maximilian Patzig, Joerg-Christian Tonn, Stefan Zausinger

**Affiliations:** 1 Department of Neurosurgery, Ludwig-Maximilians-University Munich, Marchioninistr. 15, 81377 Munich, Germany; 2 Institute of Diagnostic and Interventional Neuroradiology, Ludwig-Maximilians-University Munich, Munich, Germany

**Keywords:** Cervical myelopathy, Intraoperative sonography

## Abstract

**Background:**

The incidence of cervical myelopathy due to spinal stenosis is constantly growing in an aging population. Especially in multisegmental disease, dorsal laminectomy is the intervention of choice. Intraoperative imaging with ultrasound might provide additional information about extent and sufficiency of spinal cord decompression.

**Methods:**

In this prospective study, the width of the subarachnoid space was systematically measured by intraoperative ultrasound at predefined sites at the cranial and caudal edge of decompression in axial and sagittal reconstruction. These data were compared with corresponding sites on postoperative T2-weighted MRI imaging. In addition, the functional outcome was assessed by modified Japanese Orthopaedic Association (mJOA) score. A historical patient cohort treated without ultrasound-guided laminectomy served as control group.

**Results:**

Altogether, 29 patients were included. According to mJOA score at last follow-up, 7/29 patients reported stable symptoms and 21/29 patients showed a substantial benefit with no or minor residual neurological deficits. One patient suffered from a new C5 palsy. Intraoperative ultrasound-guided posterior decompression provided excellent overview in all cases. Measurement of the width of the subarachnoid space acquired by intraoperative ultrasound and postoperative MRI images showed a very high correlation, especially at the cranial level (*p* < 0.001, *r* = 0.880). Bland–Altman analysis showed that most patients were within the 1.96 × SD limits of agreement throughout all measurements. No ultrasound procedure-related complications were observed. Compared to a historical cohort of 27 patients, no significant differences were found regarding functional outcome (*p* = 0.711).

**Conclusion:**

Intraoperative sonography visualises the surgically achieved restoration of the subarachnoid space in good correlation with postoperative MRI and might serve as a fast, precise and reliable tool for intraoperative imaging in cervical laminectomy. However, we could not demonstrate a clinical benefit with regard to functional outcome.

## Introduction

Cervical spondylotic myelopathy (CSM) is the most common cause of cervical myelopathy [1, 14, 30]. Over the past decades, multiple techniques via an anterior and posterior approach have been established with the goal of surgical decompression to relieve the pressure on the spinal cord and re-establish circulation of the CSF. The most commonly used option of posterior decompression is laminectomy [22]; it is usually indicated in patients with cervical lordosis, ossified posterior longitudinal ligament and multilevel cervical spondylotic myelopathy as well as preserved cervical sagittal alignment and stability [7, 15, 16]. While an even greater extension of the decompression necessitates an additional instrumentation with the posterior approach, sole decompression is recommended by the WFNS Committee in its 2019 algorithm for 1- and 2-level surgery [4]. Posterior decompression of the spinal canal results in a dorsal shift; however, the impact of this shift on functional outcome is up to now discussed controversially [2, 9, 25, 29]. In MRI-controlled studies, the sole appearance of subarachnoid space surrounding the spinal cord was considered to be sufficient to secure a clinical improvement.

Intraoperative ultrasound has been validated both in spinal tumour surgery and in surgery for degenerative spine disease for optimal visualisation of the spinal cord and the surrounding subarachnoid space [8, 21, 23, 26, 28]. However, whether the visualisation of the subarachnoid space by intraoperative ultrasound correlates with the results of the postoperative MRI or the postoperative clinical course is yet unclear.

Hence, we aimed to investigate (i) the correlation of intraoperative ultrasound imaging with postoperative MRI, (ii) the association of functional outcome and intraoperative visualisation of cord decompression by ultrasound and (iii) a comparison with a historical cohort (without intraoperative ultrasound) with respect to outcome and the need for additional surgery for decompression.

## Methods and materials

### Study design and inclusion criteria

**Fig. 1:**
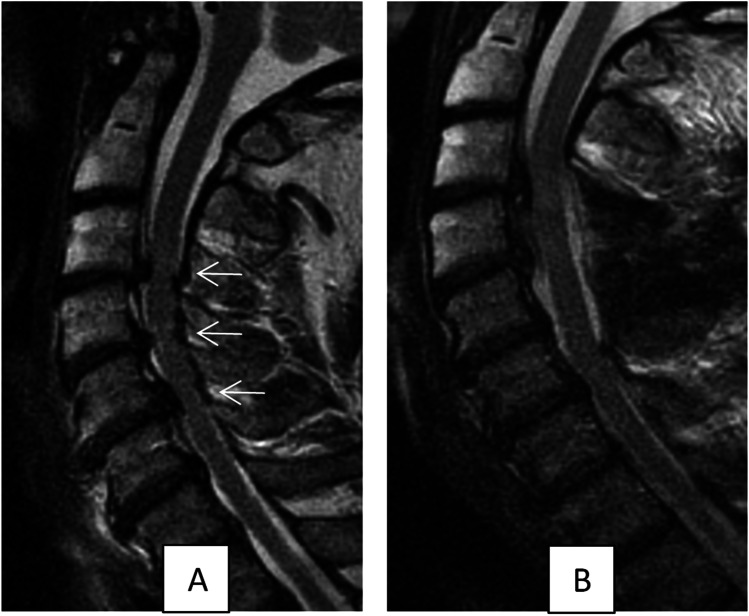
**A** Preoperative sagittal MRI (T2-weightened) showing spinal stenoses at segments C 3/4, C 4/5 and C 5/6 (arrows). **B** Postoperative MRI showing status after laminectomy C 4 and C 5 and dorsal shift of the spinal cord

After study approval by the institutional ethics board of the Ludwig-Maximilians-University (LMU) Munich, patients with one- or two-level laminectomy for cervical spinal stenosis were enrolled in this prospective study. Written consent was obtained in all patients. All patients underwent pre- and postoperative examinations including an extended evaluation of neurological symptoms of cervical myelopathy and preoperative MRI imaging of the cervical spine in supine position. Furthermore, patients with cervical spinal stenosis with fluoroscopy-guided laminectomy operated upon between 2013 and 2018 were used as a historical control cohort. Inclusion criteria consisted of (1) presence of predominantly dorsal compression of the spinal cord; (2) no signs of macroinstability on preoperative X-ray images of the cervical spine in flexion, neutral and extension position; and (3) no previous or planned instrumentation of the respective segments (Fig. 1).

### Recruitment

**Fig. 2:**
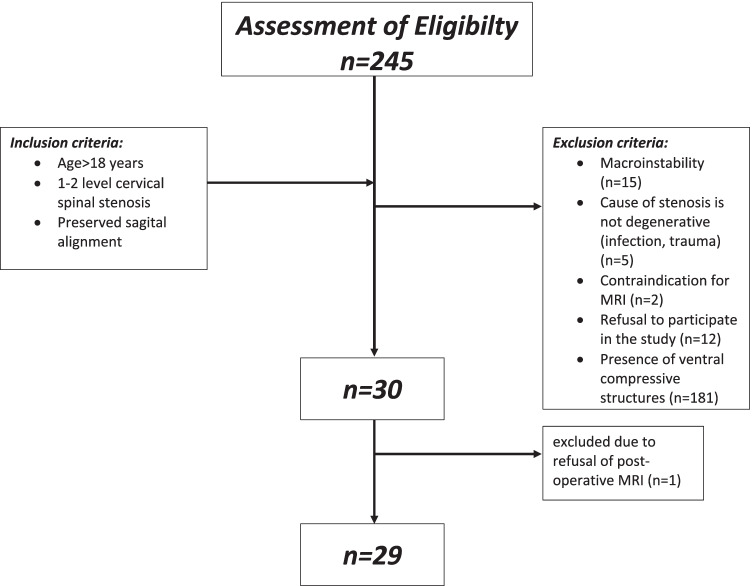
Flow chart of patient selection for enrollment in the study

Potential candidates were screened on admission. The study protocol is illustrated in Fig. 2.

### Surgical technique

Total intravenous anaesthesia was induced and patients were placed in prone position, followed by head fixation in a 3-pin Mayfield head holder. The skin incision was marked with lateral C-armed X-ray. The nuchal fascia was divided in line with the midline skin incision. After muscular dissection on both sides of the spinous process, the bone resection was completed by drilling through the lamina to the epidural space on both sides. Hypertrophic cervical ligamentum flavum was removed additionally to secure sufficient decompression under the microscope. The decompression procedure was finished with usual optical and tactical controls over the complete convexity of the dural sac laterally and craniocaudally. After these ultrasound controls were done using an ultrasound transducer probe (USt-9120, Hitachi Aloka Medical America, Wallingford, CT) with a 4–10 MHz mode setting to assess the degree of decompression in axial and sagittal orientation, to optimise the image quality, the surgical area was filled with saline. In case of ultrasonic display of remaining compression, the spinal cord decompression was resumed until display of free subarachnoid space around the spinal cord over the whole distance of decompression. The last measurement after completion of the decompression phase was used for analysis. In the historical patient cohort without ultrasound control, lateral C-armed X-ray was performed for final control of the craniocaudal extent of dorsal decompression.

### Intraoperative ultrasound

**Fig. 3:**
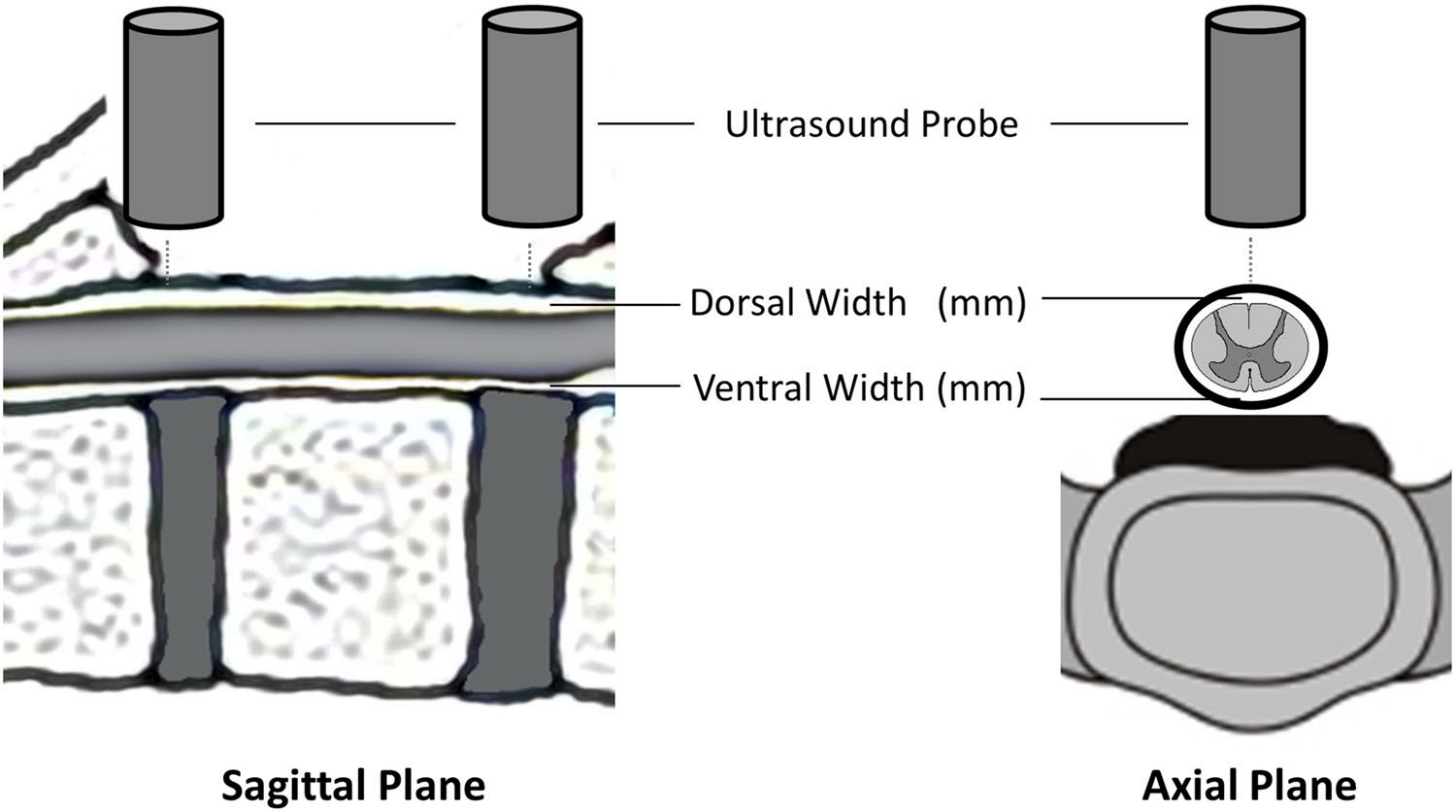
Locations of measurements of the diameters of the subarachnoid space by intraoperative ultrasonography

**Fig. 4:**
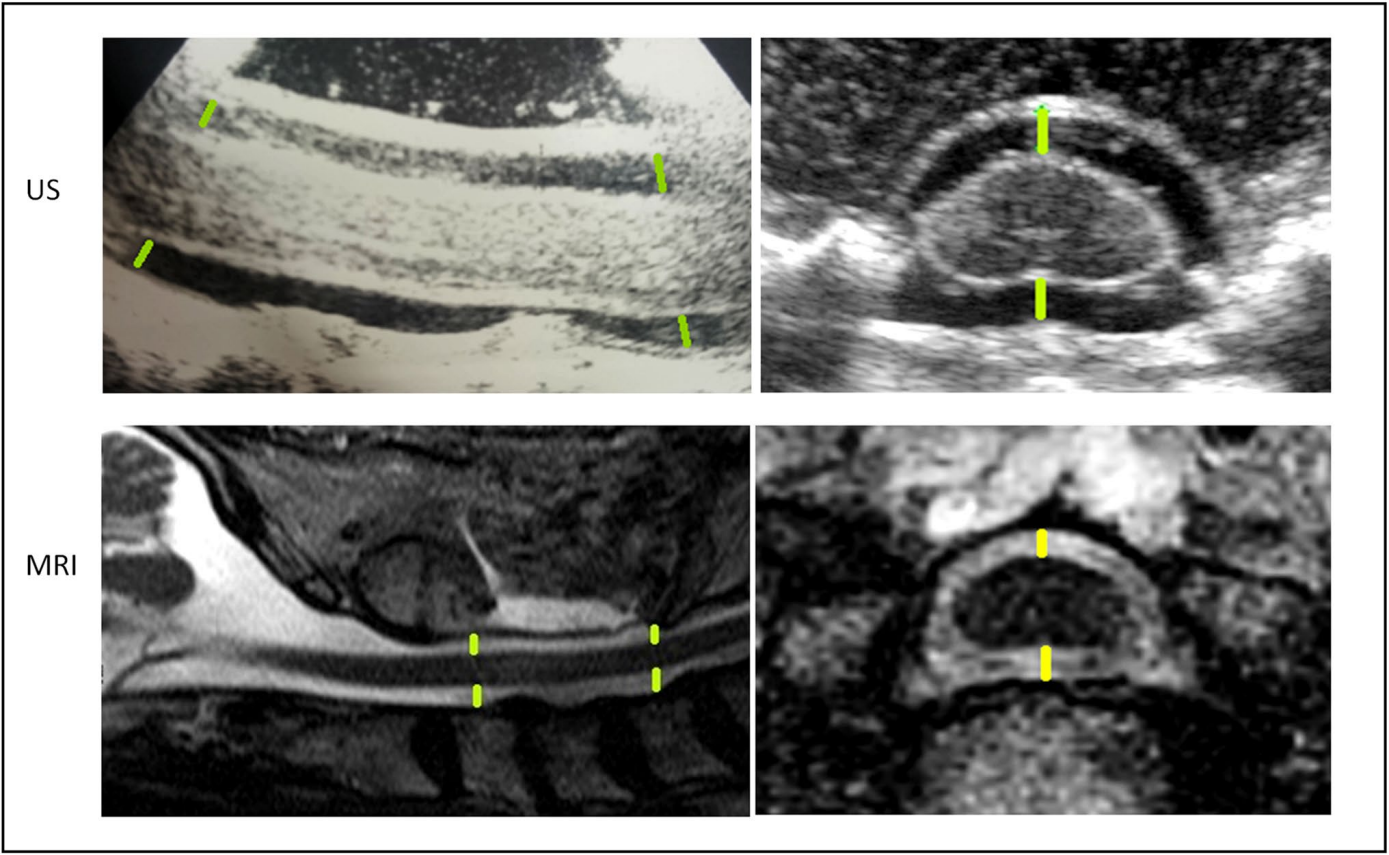
Upper line: ultrasonography of the spinal cord in sagittal and axial plane. Lower line: sagittal and axial postoperative MRI (T2-weightened). Locations of measurements are inserted in green

Ultrasound measurements were obtained by the neurosurgeon in charge for this procedure with one neurosurgeon (MF) attending all operations. The following measurements were acquired at the cranial and caudal endpoint of the decompression: (i) distance between the inner layer of the dura and the ventral surface of the spinal cord, (ii) distance between the dorsal surface of the spinal cord and the dura (Figs. 3 and 4). At the end of the procedure, the surgeon was asked to assess the intraoperative use of the ultrasound in terms of surgical workflow and guidance for decompression.

### MRI evaluation

Twenty-nine patients underwent MRI scanning within the postoperative hospitalisation period to assess the degree and adequacy of spinal cord decompression. An experienced neuroradiologist was blinded for analysis (M.P.). MRI protocols (1.5 T Magnetom Symphony, Siemens) included routine sagittal T1-weighted spin echo sequences as well as sagittal and axial T2-weighted fast spin echo sequences. The slice thickness was set at 3 mm for each sequence. Analogous to sonography, the width of the ventral and dorsal subarachnoid space at the cranial and caudal endpoint of decompression was measured on the sagittal and axial T2-weightend sequences by a neuroradiologist (MP) being blinded for the clinical course and the intraoperatively acquired data.

### Follow-up

For each patient, the modified Japanese Orthopaedic Association score (mJOA) for cervical myelopathy and McCormick score were recorded before and after surgery for neurological assessment with regard to myelopathic symptoms. Recovery rate was calculated with preoperative scores and those at final examination [27].

### Statistical analysis

The values are presented as medians and standard deviations (SD). The patient population was described with summary statistics. For comparisons for differences between image modalities and cohorts, the Student’s *t* test was used for numeric values, Mann–Whitney rank sum test for ordinal variables and *χ*^2^ test or Friedman test for nominal variables. Pearson’s correlation coefficient test and Spearman’s correlation coefficient rank test were used. Inter-modality variability was illustrated using Bland–Altman plots, where differences between measurements acquired were against the mean, presenting the mean difference and the lower and upper limits of agreement given by mean ± SD × 1.96. A *P* value < 0.05 was considered statistically significant. Analyses were performed using SPSS version 26.0 software.

## Results

### Study population

Altogether, twenty-nine patients were enrolled and underwent ultrasound-guided laminectomy at our institution. The age ranged from 46 to 85 years. The median age was 73 years (SD: 9.6 years). There were thirteen females and sixteen males. The mean duration of clinical follow-up was 3 months (range: 1–38). One patient suffered from a mild yet persisting paresis of the deltoids’ muscle; re-operation was required in two (6%) patients: in one patient due to an epidural hematoma and in one, our first patient, due to insufficient decompression.

A total of 27 patients were included in the historical group undergoing fluoroscopy-guided laminectomy. There were eleven female and sixteen male patients included with a median age 76.4 years (SD: 9.5 years). No statistical differences could be detected between both groups concerning age, gender and location of the laminectomy.

Demographic data and clinical information are summarised in Table 1.

**Table 1:** Patients’ characteristics and functional status

	US-guided group	Historical group	*P* =
Total	29	27	
Median age years (range)	71.5 (46–85)	76.4 (50–92)	0.104
Gender Male Female	16 13	16 11	0.757
Number of levels 1 Level 2 Levels	26 3	25 2	0.7
Myelomalacia	22	23	0.38
mJOA at last FU median (range)	13 (10–15)	13 (8–17)	0.711

### Imaging findings

On the axial plane, sonography displayed a diameter of the subdural space at the cranial edge of median 1.9 mm (0.9–6.2 mm, ventral side of the spinal cord) and 2.1 mm (1–5.1 mm, dorsal) compared to 1.6 mm (0–8 mm, ventral) and 1.4 mm (0.5–3.7 mm, dorsal) on the MRI scan. The findings obtained by sonography at the caudal edge were 1.7 mm (0.6–5.1 mm, ventral) and 2.1 mm (0.7–3.7 mm, dorsal) compared to 1.4 mm (0–4.6 mm, ventral) and 1.4 mm (0–5.7 mm, dorsal) on the MRI scan. On the sagittal plane level, sonographic imaging showed an extent of decompression at the cranial site of 2 mm (0.9–3.3 mm, ventral) and 2.2 mm (0.8–3.1 mm, dorsal) compared to 2 mm (0–7.2 mm, ventral) and 2.2 mm (0–3.6 mm, dorsal) on the MRI scan. At the caudal site, ultrasound showed a decompression of 2.1 mm (0.5–5.8 mm, ventral) and 2.2 mm (0.5–4.7 mm, dorsal) in comparison to 1.5 mm (0–3.8 mm, ventral) and 2.3 mm (0–6.2 mm, dorsal). Measurements of imaging are summarised in Table 2.

**Table 2:** Measurement results of width of the subarachnoid space in intraoperative US and postoperative MRI. Comparisons with Pearson correlation

	Ultrasonography	MRI	*p* =	*r* =
	Median (mm)	Median (mm)		
Axial ventral cranial	1.6	1.65	** < 0.001**	0.880
Axial dorsal cranial	2.05	1.4	0.299	0.221
Axial ventral caudal	1.7	1.4	**0.003**	0.564
Axial dorsal caudal	2.05	1.4	**0.01**	0.508
Sagittal ventral cranial	2.0	1.9	**0.008**	0.520
Sagittal dorsal cranial	2.2	1.7	**0.01**	0.5
Sagittal ventral caudal	2.0	1.4	**0.001**	0.616
Sagittal dorsal caudal	2.0	1.7	0.42	0.409

Bolded values are significant

Patients with stable/worsened neurological status showed a total decompression (ventral + dorsal subarachnoid space) in US on the caudal edge of 2.16 mm (0.68–3.9 mm) and on the cranial edge 2.12 mm (1.32–4.05 mm). Patients with improved neurological function showed a total decompression in US on the caudal edge of 2.16 mm (1.30–2.82 mm) and on the cranial edge 2.01 mm (1.35–3.35 mm). No significant difference could be detected (*p* = 0.944 and *p* = 0.101 respectively).

**Fig. 5:**
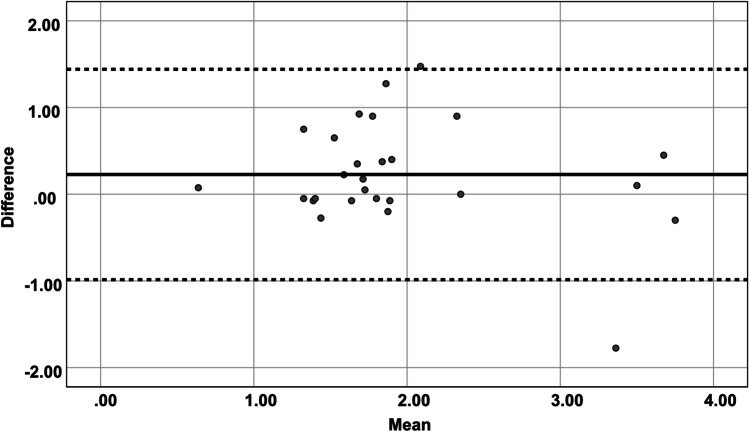
Bland–Altman plot: agreement between cranial total measurements of subarachnoid space in US and postoperative MRI

**Fig. 6:**
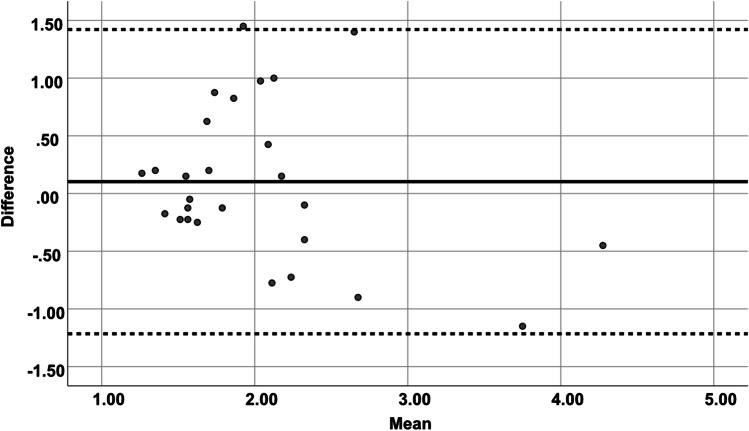
Bland–Altman plot: agreement between caudal total measurements of subarachnoid space in US and postoperative MRI

Our comparison of intraoperative ultrasound and postoperative MRI imaging showed a good concordance of the measurements recorded between the inner layer of the dura and the surface, especially at the ventral side of the spinal cord. The *r* value showed a strong correlation of the diameters of the subarachnoid space obtained by intraoperative ultrasound and postoperative MRI imaging in almost all planes, mostly in cranial axial plane (*p* < 0.001, *r* = 0.880). Bland–Altman analysis for intraoperative ultrasound showed good agreement compared with those for postoperative MRI for measurements of the subarachnoid space in the cranial and caudal edges of the laminectomy (Figs. 5 and 6). Measurements recorded in MRI images tended to show a narrower subarachnoid space.

As result of the postoperative survey, the surgeons reported that repetitive use of intraoperative ultrasound did not interrupt the surgical work flow and led to further decompression in some cases of residual stenosis visualised by sonography.

### Functional outcome

**Fig. 7:**
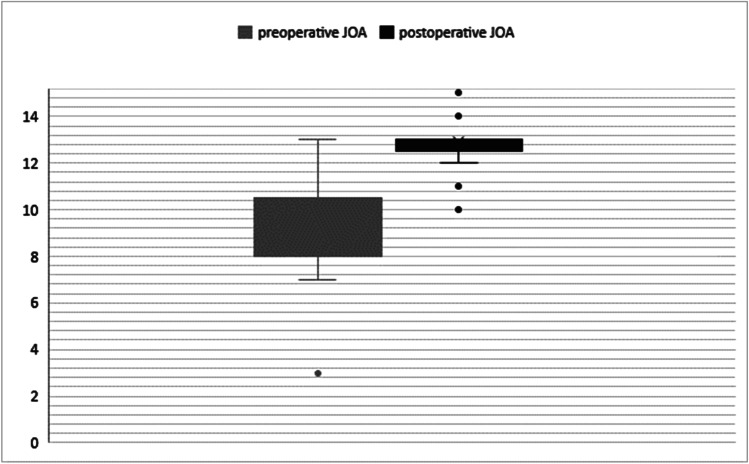
Box plot showing significant improvement of postoperative vs. preoperative mJOA scores (Friedman test)

Median preoperative mJOA Score was 10, with a range of 3 to 13. The postoperatively assessed median mJOA score was 13, ranging from 10 to 15. Functional outcome with regard to the mJOA was significantly improved postoperatively (*p* < 0.001 and *p* < 0.001, respectively). According to the Odom score at last follow-up, 7 patients (24.2%) reported stable symptoms and only one patient (3.4%) showed worsening of preoperative symptoms, with postoperative C5 palsy. Twenty-one patients (72.4%) showed a substantial benefit with no or minor residual neurological deficits. Pre- and postoperative functional status is shown in Fig. 7.

There was no statistical significant difference regarding functional outcome between our study cohort and the historical control. Clinical recovery and the width of the subarachnoid space in either imaging modality showed no significant correlation.

## Discussion

Cervical spondylotic myelopathy (CSM) resulting from pathological degeneration of the facet joints, disc herniations, hypertrophy and buckling of the ligamentum flavum or spondylosis is a common disease of the elderly becoming more and more important in an aging society. A laminectomy is often preferred in case of preserved lordosis, especially for multilevel stenosis, with a high probability of neurological recovery [4, 10]. The extent of decompression as measured by the width of the subarachnoid space anterior to the spinal cord on postoperative MRI has been postulated to be associated with favourable neurological recovery [2, 9, 17, 25, 29].

Previous studies already reported the feasibility to visualise the spinal cord with sonography [11, 12, 19, 20, 24]. Here, we performed a prospective quantitative comparison between intraoperative ultrasound and postoperative MRI. This analysis showed an excellent correlation between the intraoperative quantifications of the width of the subarachnoid space and the respective postoperative measurements in MRI. This was true for both the orientation of the plane (axial or sagittal) and the localisation site of the decompression (cranial or caudal). Hence, intraoperative sonography can indeed be used to reliably quantify the extent of cord decompression.

However, in our series, functional outcome was not associated with the width of the subarachnoid space in either imaging modality. Functional recovery after decompression of the spinal cord might be influenced by multiple factors such as extent and duration if a structural damage of the spinal cord or impairment of its microcirculation. Furthermore, one could speculate that the sole appearance of the subarachnoid space around the spinal cord might be a sufficient indicator for adequate decompression, independent of its width [29]. One of our patients showed worsening of clinical function due to C5 palsy, which is reported to occur in approximately 10% of patients [13]. This confounded the postoperative mJOA of that patient but was due to a nerve root avulsion and not related to insufficient decompression.

Another aim of our study was a comparison with a historical cohort (without intraoperative ultrasound) with respect to outcome and the need for additional surgery for decompression. We found no statistical significant difference regarding functional outcome between our study cohort and the historical control. This might be explained by a high expertise of the surgeons involved since sufficiency of the decompression can also be verified visually and haptically by experienced spine surgeons. In the first patient of our sonography cohort, a revision surgery due to incomplete decompression was necessary due to residual compression on the cranial edge of the laminectomy and unchanged postoperative functional status. This residual stenosis was only detected due to the per protocol MRI and would not have been indicated by clinical symptoms only. Nevertheless, this revision case shows that ultrasound might provoke a false sense of security. As consequence and learning effect, we paid more attention on the technique of tilting the sonography transducer head underneath the edges of the laminae adjacent to the decompression. In the control group residual postoperative stenosis in patients presenting with an unchanged neurological status cannot be ruled out since postoperative MRI were not routinely performed in this cohort. Hence, based on comparable neurological outcome, the rate of reoperations due to residual stenosis was very low in both cohorts but with a potential bias due to lack of imaging validation of decompression in the historical control group.

Limitations of the study are mainly owed to the small sample size and the lack of postoperative MRI in the historical control group. Moreover, there are a number of prognostic factors that are not reflected in the used scaling system for the evaluation of functional outcome such as age, long-term CSM symptoms, smoking and long-standing diabetes mellitus [3, 18, 31]. Variations of echogenicity within the spinal cord, which were reported to correlate with functional outcome, were not examined within the scope of the study protocol [5, 6]. The information derived from the survey among the participating surgeons is restrained since we neither documented the number of intraoperative ultrasound applications and nor the extent of additional decompression related hereto.

## Conclusion

Intraoperative sonography visualises the surgically achieved restoration of the subarachnoid space in good correlation with postoperative MRI and might serve as a fast, precise and reliable tool for intraoperative imaging in cervical laminectomy. However, we could not demonstrate a clinical benefit with regard to functional outcome.
